# Supplementary material for: Local occurrence and fast spread of B.1.1.7 lineage: A glimpse into Friuli Venezia Giulia
Source: PLoS One. 2021 Dec 14;16(12):e0261229. doi: 10.1371/journal.pone.0261229 (PMC8670677; doi:10.1371/journal.pone.0261229)
Supplement: S1 Table — (DOCX) [file pone.0261229.s001.docx]

**S1 Table.** Approximate correlation between the major SARS-CoV-2 PANGO lineage, GISAID Nextstrain clade and WHO nomenclature.

| **Pango Lineage** | **GISAID Clade** | **Nextstrain Clade** | **WHO nomenclature** | **Other Names** | **Defining Spike mutations** |
| --- | --- | --- | --- | --- | --- |
| B.1.177 | GV | 20E.EU1 |  |  | p.A222V  p.D614G |
| B.1.160 | GH | 20A.EU2 |  |  | p.S477N  p.D614G |
| B.1.1.7 | GRY | 20I | Alpha | VOC 202012/01 | p.H69_V70del p.Y144del p.N501Y p.A570D p.D614G p.P681H p.T716I p.S982A p.D1118H |
| B.1.351 | GH | 20H | Beta | VOC 202012/02 | p.D80A p.D215G p.L241_A243del p.K417N p.E484K p.N501Y p.D614G p.A701V |
| P.1 | GR | 20J | Gamma | VOC 202101/02 | p.L18F p.T20N p.P26S p.D138Y p.R190S p.K417T p.E484K p.N501Y p.D614G p.H655Y p.T1027I p.V1176F |
| P.2 | GR | 20B/S.484K |  | VUI 20201-01 | p.E484K  p.D614G  p. V1176F |
| B.1.427, B.1.429 | GH | 20C | Epsilon | CAL.20C | p.S13I p.W152C p.L452R p.D614G |
| B.1.525 | G | 21D | Eta | VUI 202102-03 | p.Q52R p.A67V p.H69_V70del p.Y144del p.E484K p.D614G p.Q677H p.F888L |
| B.1.526 | GH | 21F | Iota |  | p.L5F p.T95I p.D253G p.E484K p.D614G p.A701V |
| B.1.617.1 | G | 21B | Kappa |  | p.E154K p.L452R p.E484Q p.D614G p.P681R p.Q1071H |
| B.1.617.2 | G | 21A | Delta |  | p.T19R p.E156_F157del p.R158G p.L452R p.T478K p.D614G p.P681R p.D950N |
| B.1.258 | G | 20A/S:439K |  |  | p.S439K  p.D614G |
| B.1.221 | G | 20A/S:98F |  |  | p.S98F  p.D614G |
| B.1.1.318 | GR | 20B |  | VUI 202102/04 | p.T95I  p.Y144del  p.E484K  p.D614G  p.P681H  p.D796H |
