# Supplementary material for: Local occurrence and fast spread of B.1.1.7 lineage: A glimpse into Friuli Venezia Giulia
Source: PLoS One. 2021 Dec 14;16(12):e0261229. doi: 10.1371/journal.pone.0261229 (PMC8670677; doi:10.1371/journal.pone.0261229)
Supplement: S2 Table — (DOCX) [file pone.0261229.s002.docx]

**S2 Table.** Accession ID and virus names of the 479 sequences uploaded in the GISAID database.

| **Accession ID** | **Virus name** |
| --- | --- |
| EPI_ISL_4533464 | hCoV-19/Italy/FVG-UD-123070/2021 |
| EPI_ISL_4533465 | hCoV-19/Italy/FVG-UD-123094/2021 |
| EPI_ISL_4533466 | hCoV-19/Italy/FVG-UD-123100/2021 |
| EPI_ISL_4533467 | hCoV-19/Italy/FVG-UD-123117/2021 |
| EPI_ISL_4533468 | hCoV-19/Italy/FVG-UD-123121/2021 |
| EPI_ISL_4533469 | hCoV-19/Italy/FVG-UD-123125/2021 |
| EPI_ISL_4533470 | hCoV-19/Italy/FVG-UD-123132/2021 |
| EPI_ISL_4533471 | hCoV-19/Italy/FVG-UD-123141/2021 |
| EPI_ISL_4533472 | hCoV-19/Italy/FVG-UD-123149/2021 |
| EPI_ISL_4533473 | hCoV-19/Italy/FVG-UD-123162/2021 |
| EPI_ISL_4533474 | hCoV-19/Italy/FVG-UD-123179/2021 |
| EPI_ISL_4533475 | hCoV-19/Italy/FVG-UD-123184/2021 |
| EPI_ISL_4533476 | hCoV-19/Italy/FVG-UD-123195/2021 |
| EPI_ISL_4533478 | hCoV-19/Italy/FVG-UD-123203/2021 |
| EPI_ISL_4533479 | hCoV-19/Italy/FVG-UD-123204/2021 |
| EPI_ISL_4533480 | hCoV-19/Italy/FVG-UD-123207/2021 |
| EPI_ISL_4533481 | hCoV-19/Italy/FVG-UD-123212/2021 |
| EPI_ISL_4533482 | hCoV-19/Italy/FVG-UD-123270/2021 |
| EPI_ISL_4533483 | hCoV-19/Italy/FVG-UD-123280/2021 |
| EPI_ISL_4533484 | hCoV-19/Italy/FVG-UD-123281/2021 |
| EPI_ISL_4533485 | hCoV-19/Italy/FVG-UD-123285/2021 |
| EPI_ISL_4533486 | hCoV-19/Italy/FVG-UD-123294/2021 |
| EPI_ISL_4533487 | hCoV-19/Italy/FVG-UD-123296/2021 |
| EPI_ISL_4533492 | hCoV-19/Italy/FVG-UD-123374/2021 |
| EPI_ISL_4533493 | hCoV-19/Italy/FVG-UD-123375/2021 |
| EPI_ISL_4533494 | hCoV-19/Italy/FVG-UD-123378/2021 |
| EPI_ISL_4533495 | hCoV-19/Italy/FVG-UD-124400/2021 |
| EPI_ISL_4533497 | hCoV-19/Italy/FVG-UD-123600/2021 |
| EPI_ISL_4533498 | hCoV-19/Italy/FVG-UD-123603/2021 |
| EPI_ISL_4533499 | hCoV-19/Italy/FVG-UD-123604/2021 |
| EPI_ISL_4533501 | hCoV-19/Italy/FVG-UD-123611/2021 |
| EPI_ISL_4533501 | hCoV-19/Italy/FVG-UD-123611/2021 |
| EPI_ISL_4533503 | hCoV-19/Italy/FVG-UD-123625/2021 |
| EPI_ISL_4533504 | hCoV-19/Italy/FVG-UD-123634/2021 |
| EPI_ISL_4533505 | hCoV-19/Italy/FVG-UD-123641/2021 |
| EPI_ISL_4533507 | hCoV-19/Italy/FVG-UD-123658/2021 |
| EPI_ISL_4462121 | hCoV-19/Italy/FVG-UD-123989/2021 |
| EPI_ISL_4533511 | hCoV-19/Italy/FVG-UD-123995/2021 |
| EPI_ISL_4533512 | hCoV-19/Italy/FVG-UD-124001/2021 |
| EPI_ISL_4533513 | hCoV-19/Italy/FVG-UD-124015/2021 |
| EPI_ISL_4533515 | hCoV-19/Italy/FVG-UD-123105/2021 |
| EPI_ISL_2938317 | hCoV-19/Italy/FVG-UD-123108/2021 |
| EPI_ISL_4533516 | hCoV-19/Italy/FVG-UD-123134/2021 |
| EPI_ISL_2938318 | hCoV-19/Italy/FVG-UD-123188/2021 |
| EPI_ISL_4462122 | hCoV-19/Italy/FVG-UD-123372/2021 |
| EPI_ISL_2966240 | hCoV-19/Italy/FVG-UD-123286/2021 |
| EPI_ISL_2938319 | hCoV-19/Italy/FVG-UD-123308/2021 |
| EPI_ISL_4533519 | hCoV-19/Italy/FVG-UD-122931/2021 |
| EPI_ISL_4533520 | hCoV-19/Italy/FVG-UD-122933/2021 |
| EPI_ISL_4533521 | hCoV-19/Italy/FVG-UD-123541/2021 |
| EPI_ISL_4462124 | hCoV-19/Italy/FVG-UD-123547/2021 |
| EPI_ISL_4533524 | hCoV-19/Italy/FVG-UD-123539/2021 |
| EPI_ISL_4533525 | hCoV-19/Italy/FVG-UD-123523/2021 |
| EPI_ISL_4533526 | hCoV-19/Italy/FVG-UD-123477/2021 |
| EPI_ISL_4533527 | hCoV-19/Italy/FVG-UD-122625/2021 |
| EPI_ISL_4533528 | hCoV-19/Italy/FVG-UD-122622/2021 |
| EPI_ISL_4533529 | hCoV-19/Italy/FVG-UD-122818/2021 |
| EPI_ISL_4533530 | hCoV-19/Italy/FVG-UD-122775/2021 |
| EPI_ISL_4533534 | hCoV-19/Italy/FVG-UD-122539/2021 |
| EPI_ISL_4533477 | hCoV-19/Italy/FVG-UD-121951/2021 |
| EPI_ISL_2938314 | hCoV-19/Italy/FVG-UD-121944/2021 |
| EPI_ISL_4533531 | hCoV-19/Italy/FVG-UD-121819/2021 |
| EPI_ISL_4533532 | hCoV-19/Italy/FVG-UD-121818/2021 |
| EPI_ISL_4558193 | hCoV-19/Italy/FVG-UD-121821/2021 |
| EPI_ISL_4462125 | hCoV-19/Italy/FVG-UD-121943/2021 |
| EPI_ISL_4533533 | hCoV-19/Italy/FVG-UD-121820/2021 |
| EPI_ISL_4543122 | hCoV-19/Italy/FVG-UD-121902/2021 |
| EPI_ISL_4543123 | hCoV-19/Italy/FVG-UD-121903/2021 |
| EPI_ISL_4543131 | hCoV-19/Italy/FVG-UD-121904/2021 |
| EPI_ISL_4543124 | hCoV-19/Italy/FVG-UD-121913/2021 |
| EPI_ISL_4558574 | hCoV-19/Italy/FVG-UD-121914/2021 |
| EPI_ISL_4543125 | hCoV-19/Italy/FVG-UD-121916/2021 |
| EPI_ISL_4543126 | hCoV-19/Italy/FVG-UD-121917/2021 |
| EPI_ISL_4543127 | hCoV-19/Italy/FVG-UD-121920/2021 |
| EPI_ISL_4543134 | hCoV-19/Italy/FVG-UD-121865/2021 |
| EPI_ISL_4558575 | hCoV-19/Italy/FVG-UD-121868/2021 |
| EPI_ISL_4543128 | hCoV-19/Italy/FVG-UD-121871/2021 |
| EPI_ISL_4558192 | hCoV-19/Italy/FVG-UD-122368/2021 |
| EPI_ISL_4543135 | hCoV-19/Italy/FVG-UD-122709/2021 |
| EPI_ISL_4543120 | hCoV-19/Italy/FVG-UD-122735/2021 |
| EPI_ISL_4543136 | hCoV-19/Italy/FVG-UD-122726/2021 |
| EPI_ISL_4558190 | hCoV-19/Italy/FVG-UD-122714/2021 |
| EPI_ISL_4543137 | hCoV-19/Italy/FVG-UD-122739/2021 |
| EPI_ISL_4543119 | hCoV-19/Italy/FVG-UD-122600/2021 |
| EPI_ISL_4558573 | hCoV-19/Italy/FVG-UD-122642/2021 |
| EPI_ISL_4543138 | hCoV-19/Italy/FVG-UD-122651/2021 |
| EPI_ISL_4543118 | hCoV-19/Italy/FVG-UD-122655/2021 |
| EPI_ISL_4543132 | hCoV-19/Italy/FVG-UD-122936/2021 |
| EPI_ISL_4558191 | hCoV-19/Italy/FVG-UD-123199/2021 |
| EPI_ISL_2966237 | hCoV-19/Italy/FVG-UD-122275/2021 |
| EPI_ISL_4543133 | hCoV-19/Italy/FVG-UD-123775/2021 |
| EPI_ISL_4543139 | hCoV-19/Italy/FVG-UD-122681/2021 |
| EPI_ISL_2966238 | hCoV-19/Italy/FVG-UD-122641/2021 |
| EPI_ISL_2966239 | hCoV-19/Italy/FVG-UD-122657/2021 |
| EPI_ISL_4558194 | hCoV-19/Italy/FVG-UD-122890/2021 |
| EPI_ISL_4543140 | hCoV-19/Italy/FVG-UD-121866/2021 |
| EPI_ISL_4543144 | hCoV-19/Italy/FVG-UD-122618/2021 |
| EPI_ISL_4543121 | hCoV-19/Italy/FVG-UD-124237/2021 |
| EPI_ISL_4543141 | hCoV-19/Italy/FVG-UD-121814/2021 |
| EPI_ISL_4543129 | hCoV-19/Italy/FVG-UD-122471/2021 |
| EPI_ISL_4543143 | hCoV-19/Italy/FVG-UD-122935/2021 |
| EPI_ISL_4543142 | hCoV-19/Italy/FVG-UD-124220/2021 |
| EPI_ISL_4543130 | hCoV-19/Italy/FVG-UD-124243/2021 |
| EPI_ISL_4462127 | hCoV-19/Italy/FVG-UD-8990/2021 |
| EPI_ISL_2938320 | hCoV-19/Italy/FVG-UD-8406/2021 |
| EPI_ISL_4462128 | hCoV-19/Italy/FVG-UD-8549/2021 |
| EPI_ISL_4462129 | hCoV-19/Italy/FVG-UD-8401/2021 |
| EPI_ISL_4462130 | hCoV-19/Italy/FVG-UD-8385/2021 |
| EPI_ISL_4533535 | hCoV-19/Italy/FVG-UD-8431/2021 |
| EPI_ISL_4462131 | hCoV-19/Italy/FVG-UD-8526/2021 |
| EPI_ISL_4462132 | hCoV-19/Italy/FVG-UD-8428/2021 |
| EPI_ISL_2966241 | hCoV-19/Italy/FVG-UD-9958/2021 |
| EPI_ISL_4462133 | hCoV-19/Italy/FVG-UD-8449/2021 |
| EPI_ISL_4462134 | hCoV-19/Italy/FVG-UD-8447/2021 |
| EPI_ISL_4462135 | hCoV-19/Italy/FVG-UD-8906/2021 |
| EPI_ISL_4462136 | hCoV-19/Italy/FVG-UD-8469/2021 |
| EPI_ISL_4462137 | hCoV-19/Italy/FVG-UD-8463/2021 |
| EPI_ISL_4462138 | hCoV-19/Italy/FVG-UD-8900/2021 |
| EPI_ISL_2938321 | hCoV-19/Italy/FVG-UD-8394/2021 |
| EPI_ISL_4462139 | hCoV-19/Italy/FVG-UD-9844/2021 |
| EPI_ISL_4462140 | hCoV-19/Italy/FVG-UD-8905/2021 |
| EPI_ISL_4462141 | hCoV-19/Italy/FVG-UD-8378/2021 |
| EPI_ISL_4462142 | hCoV-19/Italy/FVG-UD-8904/2021 |
| EPI_ISL_4462143 | hCoV-19/Italy/FVG-UD-9934/2021 |
| EPI_ISL_4462144 | hCoV-19/Italy/FVG-UD-5081/2021 |
| EPI_ISL_4462145 | hCoV-19/Italy/FVG-UD-7577/2021 |
| EPI_ISL_4462146 | hCoV-19/Italy/FVG-UD-8422/2021 |
| EPI_ISL_2938322 | hCoV-19/Italy/FVG-UD-8263/2021 |
| EPI_ISL_4462147 | hCoV-19/Italy/FVG-UD-8256/2021 |
| EPI_ISL_4462148 | hCoV-19/Italy/FVG-UD-6070/2021 |
| EPI_ISL_4462149 | hCoV-19/Italy/FVG-UD-6097/2021 |
| EPI_ISL_4462150 | hCoV-19/Italy/FVG-UD-8913/2021 |
| EPI_ISL_4462151 | hCoV-19/Italy/FVG-UD-8277/2021 |
| EPI_ISL_4462152 | hCoV-19/Italy/FVG-UD-7565/2021 |
| EPI_ISL_4462153 | hCoV-19/Italy/FVG-UD-7695/2021 |
| EPI_ISL_4462154 | hCoV-19/Italy/FVG-UD-10605/2021 |
| EPI_ISL_4533536 | hCoV-19/Italy/FVG-UD-9990/2021 |
| EPI_ISL_4462155 | hCoV-19/Italy/FVG-UD-6101/2021 |
| EPI_ISL_4533537 | hCoV-19/Italy/FVG-UD-9989/2021 |
| EPI_ISL_4462156 | hCoV-19/Italy/FVG-UD-8592/2021 |
| EPI_ISL_4462157 | hCoV-19/Italy/FVG-UD-8421/2021 |
| EPI_ISL_2966242 | hCoV-19/Italy/FVG-UD-3221/2021 |
| EPI_ISL_4462158 | hCoV-19/Italy/FVG-UD-18321/2021 |
| EPI_ISL_4462159 | hCoV-19/Italy/FVG-UD-16904/2021 |
| EPI_ISL_4462160 | hCoV-19/Italy/FVG-UD-16900/2021 |
| EPI_ISL_4462161 | hCoV-19/Italy/FVG-UD-16902/2021 |
| EPI_ISL_4462162 | hCoV-19/Italy/FVG-UD-4270/2021 |
| EPI_ISL_2938324 | hCoV-19/Italy/FVG-UD-6098/2021 |
| EPI_ISL_2966243 | hCoV-19/Italy/FVG-UD-10629/2021 |
| EPI_ISL_2966244 | hCoV-19/Italy/FVG-UD-7423/2021 |
| EPI_ISL_4462163 | hCoV-19/Italy/FVG-UD-4208/2021 |
| EPI_ISL_4462164 | hCoV-19/Italy/FVG-UD-4379/2021 |
| EPI_ISL_4462165 | hCoV-19/Italy/FVG-UD-4272/2021 |
| EPI_ISL_4462166 | hCoV-19/Italy/FVG-UD-4227/2021 |
| EPI_ISL_4462167 | hCoV-19/Italy/FVG-UD-4377/2021 |
| EPI_ISL_4462168 | hCoV-19/Italy/FVG-UD-4372/2021 |
| EPI_ISL_4462169 | hCoV-19/Italy/FVG-UD-8033/2021 |
| EPI_ISL_4462170 | hCoV-19/Italy/FVG-UD-7409/2021 |
| EPI_ISL_4462171 | hCoV-19/Italy/FVG-UD-8066/2021 |
| EPI_ISL_4462172 | hCoV-19/Italy/FVG-UD-7407/2021 |
| EPI_ISL_4462173 | hCoV-19/Italy/FVG-UD-4196/2021 |
| EPI_ISL_4462174 | hCoV-19/Italy/FVG-UD-4267/2021 |
| EPI_ISL_4462175 | hCoV-19/Italy/FVG-UD-7744/2021 |
| EPI_ISL_2966245 | hCoV-19/Italy/FVG-UD-4241/2021 |
| EPI_ISL_4462176 | hCoV-19/Italy/FVG-UD-4255/2021 |
| EPI_ISL_4533538 | hCoV-19/Italy/FVG-UD-7598/2021 |
| EPI_ISL_4462177 | hCoV-19/Italy/FVG-UD-4186/2021 |
| EPI_ISL_4462178 | hCoV-19/Italy/FVG-UD-8026/2021 |
| EPI_ISL_4462179 | hCoV-19/Italy/FVG-UD-8371/2021 |
| EPI_ISL_4462180 | hCoV-19/Italy/FVG-UD-24263/2021 |
| EPI_ISL_4462181 | hCoV-19/Italy/FVG-UD-24229/2021 |
| EPI_ISL_4462182 | hCoV-19/Italy/FVG-UD-24211/2021 |
| EPI_ISL_2938325 | hCoV-19/Italy/FVG-UD-24205/2021 |
| EPI_ISL_4462183 | hCoV-19/Italy/FVG-UD-24199/2021 |
| EPI_ISL_4462184 | hCoV-19/Italy/FVG-UD-23933/2021 |
| EPI_ISL_4462185 | hCoV-19/Italy/FVG-UD-28300/2021 |
| EPI_ISL_4533539 | hCoV-19/Italy/FVG-UD-142882/2021 |
| EPI_ISL_4275793 | hCoV-19/Italy/FVG-UD-39632/2021 |
| EPI_ISL_4275794 | hCoV-19/Italy/FVG-UD-42243/2021 |
| EPI_ISL_4275795 | hCoV-19/Italy/FVG-UD-42313/2021 |
| EPI_ISL_4275796 | hCoV-19/Italy/FVG-UD-42321/2021 |
| EPI_ISL_4275797 | hCoV-19/Italy/FVG-UD-42330/2021 |
| EPI_ISL_4275798 | hCoV-19/Italy/FVG-UD-42241/2021 |
| EPI_ISL_4275799 | hCoV-19/Italy/FVG-UD-42329/2021 |
| EPI_ISL_4275800 | hCoV-19/Italy/FVG-UD-42282/2021 |
| EPI_ISL_4275801 | hCoV-19/Italy/FVG-UD-40119/2021 |
| EPI_ISL_4275802 | hCoV-19/Italy/FVG-UD-37749/2021 |
| EPI_ISL_4275803 | hCoV-19/Italy/FVG-UD-42328/2021 |
| EPI_ISL_4275804 | hCoV-19/Italy/FVG-UD-37772/2021 |
| EPI_ISL_4275805 | hCoV-19/Italy/FVG-UD-38519/2021 |
| EPI_ISL_4275806 | hCoV-19/Italy/FVG-UD-39284/2021 |
| EPI_ISL_4275807 | hCoV-19/Italy/FVG-UD-39469/2021 |
| EPI_ISL_4275808 | hCoV-19/Italy/FVG-UD-49344/2021 |
| EPI_ISL_4275809 | hCoV-19/Italy/FVG-UD-39127/2021 |
| EPI_ISL_4275810 | hCoV-19/Italy/FVG-UD-39145/2021 |
| EPI_ISL_4275811 | hCoV-19/Italy/FVG-UD-40136/2021 |
| EPI_ISL_4275812 | hCoV-19/Italy/FVG-UD-39143/2021 |
| EPI_ISL_4275813 | hCoV-19/Italy/FVG-UD-39152/2021 |
| EPI_ISL_4275814 | hCoV-19/Italy/FVG-UD-39137/2021 |
| EPI_ISL_4275815 | hCoV-19/Italy/FVG-UD-39271/2021 |
| EPI_ISL_4275816 | hCoV-19/Italy/FVG-UD-40092/2021 |
| EPI_ISL_4275817 | hCoV-19/Italy/FVG-UD-39375/2021 |
| EPI_ISL_4275818 | hCoV-19/Italy/FVG-UD-39307/2021 |
| EPI_ISL_4275819 | hCoV-19/Italy/FVG-UD-39294/2021 |
| EPI_ISL_4275820 | hCoV-19/Italy/FVG-UD-39302/2021 |
| EPI_ISL_4275821 | hCoV-19/Italy/FVG-UD-44390/2021 |
| EPI_ISL_4275822 | hCoV-19/Italy/FVG-UD-42365/2021 |
| EPI_ISL_4275823 | hCoV-19/Italy/FVG-UD-39631/2021 |
| EPI_ISL_4275824 | hCoV-19/Italy/FVG-UD-42624/2021 |
| EPI_ISL_4275825 | hCoV-19/Italy/FVG-UD-38566/2021 |
| EPI_ISL_4275826 | hCoV-19/Italy/FVG-UD-42568/2021 |
| EPI_ISL_4275827 | hCoV-19/Italy/FVG-UD-42598/2021 |
| EPI_ISL_4275828 | hCoV-19/Italy/FVG-UD-42599/2021 |
| EPI_ISL_4275829 | hCoV-19/Italy/FVG-UD-45445/2021 |
| EPI_ISL_4275830 | hCoV-19/Italy/FVG-UD-33820/2021 |
| EPI_ISL_4275831 | hCoV-19/Italy/FVG-UD-45760/2021 |
| EPI_ISL_4275832 | hCoV-19/Italy/FVG-UD-35078/2021 |
| EPI_ISL_4275834 | hCoV-19/Italy/FVG-UD-45725/2021 |
| EPI_ISL_4275835 | hCoV-19/Italy/FVG-UD-45721/2021 |
| EPI_ISL_4275836 | hCoV-19/Italy/FVG-UD-33822/2021 |
| EPI_ISL_4275838 | hCoV-19/Italy/FVG-UD-38565/2021 |
| EPI_ISL_4313819 | hCoV-19/Italy/FVG-UD-13487/2021 |
| EPI_ISL_4313821 | hCoV-19/Italy/FVG-UD-42812/2021 |
| EPI_ISL_4275839 | hCoV-19/Italy/FVG-UD-40068/2021 |
| EPI_ISL_4275840 | hCoV-19/Italy/FVG-UD-39819/2021 |
| EPI_ISL_4275841 | hCoV-19/Italy/FVG-UD-41530/2021 |
| EPI_ISL_4313743 | hCoV-19/Italy/FVG-UD-41801/2021 |
| EPI_ISL_4275842 | hCoV-19/Italy/FVG-UD-45394/2021 |
| EPI_ISL_4275843 | hCoV-19/Italy/FVG-UD-45465/2021 |
| EPI_ISL_4275844 | hCoV-19/Italy/FVG-UD-42360/2021 |
| EPI_ISL_4275845 | hCoV-19/Italy/FVG-UD-42958/2021 |
| EPI_ISL_4275846 | hCoV-19/Italy/FVG-UD-42781/2021 |
| EPI_ISL_4275847 | hCoV-19/Italy/FVG-UD-42772/2021 |
| EPI_ISL_4275848 | hCoV-19/Italy/FVG-UD-44458/2021 |
| EPI_ISL_4275849 | hCoV-19/Italy/FVG-UD-42797/2021 |
| EPI_ISL_4275850 | hCoV-19/Italy/FVG-UD-39276/2021 |
| EPI_ISL_4275851 | hCoV-19/Italy/FVG-UD-40233/2021 |
| EPI_ISL_4275852 | hCoV-19/Italy/FVG-UD-38563/2021 |
| EPI_ISL_4275853 | hCoV-19/Italy/FVG-UD-38918/2021 |
| EPI_ISL_4275854 | hCoV-19/Italy/FVG-UD-42230/2021 |
| EPI_ISL_4275855 | hCoV-19/Italy/FVG-UD-45459/2021 |
| EPI_ISL_4275856 | hCoV-19/Italy/FVG-UD-38600/2021 |
| EPI_ISL_4313745 | hCoV-19/Italy/FVG-UD-34716/2021 |
| EPI_ISL_4275857 | hCoV-19/Italy/FVG-UD-44917/2021 |
| EPI_ISL_4275858 | hCoV-19/Italy/FVG-UD-34330/2021 |
| EPI_ISL_4275859 | hCoV-19/Italy/FVG-UD-38601/2021 |
| EPI_ISL_4275860 | hCoV-19/Italy/FVG-UD-35005/2021 |
| EPI_ISL_4275861 | hCoV-19/Italy/FVG-UD-34061/2021 |
| EPI_ISL_4275862 | hCoV-19/Italy/FVG-UD-34325/2021 |
| EPI_ISL_4275863 | hCoV-19/Italy/FVG-UD-37743/2021 |
| EPI_ISL_4275864 | hCoV-19/Italy/FVG-UD-42387/2021 |
| EPI_ISL_4275865 | hCoV-19/Italy/FVG-UD-44912/2021 |
| EPI_ISL_4275866 | hCoV-19/Italy/FVG-UD-42364/2021 |
| EPI_ISL_4275867 | hCoV-19/Italy/FVG-UD-42423/2021 |
| EPI_ISL_4275868 | hCoV-19/Italy/FVG-UD-44451/2021 |
| EPI_ISL_4275869 | hCoV-19/Italy/FVG-UD-42421/2021 |
| EPI_ISL_4275870 | hCoV-19/Italy/FVG-UD-42552/2021 |
| EPI_ISL_4275871 | hCoV-19/Italy/FVG-UD-41798/2021 |
| EPI_ISL_4275872 | hCoV-19/Italy/FVG-UD-40315/2021 |
| EPI_ISL_4313749 | hCoV-19/Italy/FVG-UD-189903/2021 |
| EPI_ISL_4313755 | hCoV-19/Italy/FVG-UD-184594/2021 |
| EPI_ISL_4313824 | hCoV-19/Italy/FVG-UD-184155/2021 |
| EPI_ISL_4313831 | hCoV-19/Italy/FVG-UD-184159/2021 |
| EPI_ISL_4313838 | hCoV-19/Italy/FVG-UD-184057/2021 |
| EPI_ISL_4313840 | hCoV-19/Italy/FVG-UD-184583/2021 |
| EPI_ISL_4275873 | hCoV-19/Italy/FVG-UD-190754/2021 |
| EPI_ISL_4313807 | hCoV-19/Italy/FVG-UD-182358/2021 |
| EPI_ISL_4313888 | hCoV-19/Italy/FVG-UD-182378/2021 |
| EPI_ISL_4313843 | hCoV-19/Italy/FVG-UD-182228/2021 |
| EPI_ISL_4313758 | hCoV-19/Italy/FVG-UD-182237/2021 |
| EPI_ISL_4313760 | hCoV-19/Italy/FVG-UD-184772/2021 |
| EPI_ISL_4313846 | hCoV-19/Italy/FVG-UD-147819/2021 |
| EPI_ISL_4313809 | hCoV-19/Italy/FVG-UD-182693/2021 |
| EPI_ISL_4313763 | hCoV-19/Italy/FVG-UD-182722/2021 |
| EPI_ISL_4313765 | hCoV-19/Italy/FVG-UD-181710/2021 |
| EPI_ISL_4313849 | hCoV-19/Italy/FVG-UD-184345/2021 |
| EPI_ISL_4313767 | hCoV-19/Italy/FVG-UD-184274/2021 |
| EPI_ISL_4313851 | hCoV-19/Italy/FVG-UD-184298/2021 |
| EPI_ISL_4313854 | hCoV-19/Italy/FVG-UD-182145/2021 |
| EPI_ISL_4313770 | hCoV-19/Italy/FVG-UD-182151/2021 |
| EPI_ISL_4313856 | hCoV-19/Italy/FVG-UD-182180/2021 |
| EPI_ISL_4313772 | hCoV-19/Italy/FVG-UD-184756/2021 |
| EPI_ISL_4313859 | hCoV-19/Italy/FVG-UD-184766/2021 |
| EPI_ISL_4313862 | hCoV-19/Italy/FVG-UD-184768/2021 |
| EPI_ISL_4313774 | hCoV-19/Italy/FVG-UD-184769/2021 |
| EPI_ISL_4313777 | hCoV-19/Italy/FVG-UD-184761/2021 |
| EPI_ISL_4313864 | hCoV-19/Italy/FVG-UD-190227/2021 |
| EPI_ISL_4313867 | hCoV-19/Italy/FVG-UD-185729/2021 |
| EPI_ISL_4313869 | hCoV-19/Italy/FVG-UD-185730/2021 |
| EPI_ISL_4313872 | hCoV-19/Italy/FVG-UD-187613/2021 |
| EPI_ISL_4313779 | hCoV-19/Italy/FVG-UD-191626/2021 |
| EPI_ISL_4313781 | hCoV-19/Italy/FVG-UD-188881/2021 |
| EPI_ISL_4313812 | hCoV-19/Italy/FVG-UD-190168/2021 |
| EPI_ISL_4313784 | hCoV-19/Italy/FVG-UD-208693/2021 |
| EPI_ISL_4313875 | hCoV-19/Italy/FVG-UD-191587/2021 |
| EPI_ISL_4313786 | hCoV-19/Italy/FVG-UD-187615/2021 |
| EPI_ISL_4313877 | hCoV-19/Italy/FVG-UD-191625/2021 |
| EPI_ISL_4313789 | hCoV-19/Italy/FVG-UD-188614/2021 |
| EPI_ISL_4313804 | hCoV-19/Italy/FVG-UD-185721/2021 |
| EPI_ISL_4313814 | hCoV-19/Italy/FVG-UD-190952/2021 |
| EPI_ISL_4275874 | hCoV-19/Italy/FVG-UD-189026/2021 |
| EPI_ISL_4313791 | hCoV-19/Italy/FVG-UD-189430/2021 |
| EPI_ISL_4313794 | hCoV-19/Italy/FVG-UD-189391/2021 |
| EPI_ISL_4313797 | hCoV-19/Italy/FVG-UD-189763/2021 |
| EPI_ISL_4275875 | hCoV-19/Italy/FVG-UD-188871/2021 |
| EPI_ISL_4275876 | hCoV-19/Italy/FVG-UD-8962/2021 |
| EPI_ISL_4313879 | hCoV-19/Italy/FVG-UD-189714/2021 |
| EPI_ISL_4313882 | hCoV-19/Italy/FVG-UD-190953/2021 |
| EPI_ISL_4313884 | hCoV-19/Italy/FVG-UD-190675/2021 |
| EPI_ISL_4313801 | hCoV-19/Italy/FVG-UD-190690/2021 |
| EPI_ISL_4313817 | hCoV-19/Italy/FVG-UD-188872/2021 |
| EPI_ISL_4313886 | hCoV-19/Italy/FVG-UD-191301/2021 |
| EPI_ISL_4301008 | hCoV-19/Italy/FVG-UD-64713/2021 |
| EPI_ISL_4301009 | hCoV-19/Italy/FVG-UD-64714/2021 |
| EPI_ISL_4301010 | hCoV-19/Italy/FVG-UD-72496/2021 |
| EPI_ISL_4301011 | hCoV-19/Italy/FVG-UD-73660/2021 |
| EPI_ISL_4301012 | hCoV-19/Italy/FVG-UD-60998/2021 |
| EPI_ISL_4301013 | hCoV-19/Italy/FVG-UD-62448/2021 |
| EPI_ISL_4301014 | hCoV-19/Italy/FVG-UD-62436/2021 |
| EPI_ISL_4301015 | hCoV-19/Italy/FVG-UD-70341/2021 |
| EPI_ISL_4301016 | hCoV-19/Italy/FVG-UD-70327/2021 |
| EPI_ISL_4301017 | hCoV-19/Italy/FVG-UD-73663/2021 |
| EPI_ISL_4301018 | hCoV-19/Italy/FVG-UD-73665/2021 |
| EPI_ISL_4301019 | hCoV-19/Italy/FVG-UD-73662/2021 |
| EPI_ISL_4301020 | hCoV-19/Italy/FVG-UD-62450/2021 |
| EPI_ISL_4301021 | hCoV-19/Italy/FVG-UD-63830/2021 |
| EPI_ISL_4301022 | hCoV-19/Italy/FVG-UD-73575/2021 |
| EPI_ISL_4301023 | hCoV-19/Italy/FVG-UD-74001/2021 |
| EPI_ISL_4301024 | hCoV-19/Italy/FVG-UD-73454/2021 |
| EPI_ISL_4301025 | hCoV-19/Italy/FVG-UD-69779/2021 |
| EPI_ISL_4301026 | hCoV-19/Italy/FVG-UD-69778/2021 |
| EPI_ISL_4301027 | hCoV-19/Italy/FVG-UD-74214/2021 |
| EPI_ISL_4301028 | hCoV-19/Italy/FVG-UD-61242/2021 |
| EPI_ISL_4301029 | hCoV-19/Italy/FVG-UD-73888/2021 |
| EPI_ISL_4301030 | hCoV-19/Italy/FVG-UD-74003/2021 |
| EPI_ISL_4301031 | hCoV-19/Italy/FVG-UD-75237/2021 |
| EPI_ISL_4301032 | hCoV-19/Italy/FVG-UD-48207/2021 |
| EPI_ISL_4301033 | hCoV-19/Italy/FVG-UD-63809/2021 |
| EPI_ISL_4301034 | hCoV-19/Italy/FVG-UD-65152/2021 |
| EPI_ISL_4301035 | hCoV-19/Italy/FVG-UD-73268/2021 |
| EPI_ISL_4301036 | hCoV-19/Italy/FVG-UD-73604/2021 |
| EPI_ISL_4301037 | hCoV-19/Italy/FVG-UD-69427/2021 |
| EPI_ISL_4301038 | hCoV-19/Italy/FVG-UD-68190/2021 |
| EPI_ISL_4301039 | hCoV-19/Italy/FVG-UD-71548/2021 |
| EPI_ISL_4301040 | hCoV-19/Italy/FVG-UD-69744/2021 |
| EPI_ISL_4301041 | hCoV-19/Italy/FVG-UD-192655/2021 |
| EPI_ISL_4301042 | hCoV-19/Italy/FVG-UD-109583/2021 |
| EPI_ISL_4301043 | hCoV-19/Italy/FVG-UD-107432/2021 |
| EPI_ISL_4301044 | hCoV-19/Italy/FVG-UD-90440/2021 |
| EPI_ISL_4301045 | hCoV-19/Italy/FVG-UD-106592/2021 |
| EPI_ISL_4301046 | hCoV-19/Italy/FVG-UD-108562/2021 |
| EPI_ISL_4301047 | hCoV-19/Italy/FVG-UD-103767/2021 |
| EPI_ISL_4301048 | hCoV-19/Italy/FVG-UD-103327/2021 |
| EPI_ISL_4301049 | hCoV-19/Italy/FVG-UD-207737/2021 |
| EPI_ISL_4301050 | hCoV-19/Italy/FVG-UD-201493/2021 |
| EPI_ISL_4301051 | hCoV-19/Italy/FVG-UD-201469/2021 |
| EPI_ISL_4301052 | hCoV-19/Italy/FVG-UD-201468/2021 |
| EPI_ISL_4301053 | hCoV-19/Italy/FVG-UD-225422/2021 |
| EPI_ISL_4301054 | hCoV-19/Italy/FVG-UD-204859/2021 |
| EPI_ISL_4301055 | hCoV-19/Italy/FVG-UD-201416/2021 |
| EPI_ISL_4301056 | hCoV-19/Italy/FVG-UD-201412/2021 |
| EPI_ISL_4301057 | hCoV-19/Italy/FVG-UD-207574/2021 |
| EPI_ISL_4301058 | hCoV-19/Italy/FVG-UD-201411/2021 |
| EPI_ISL_4301059 | hCoV-19/Italy/FVG-UD-162982/2021 |
| EPI_ISL_4301060 | hCoV-19/Italy/FVG-UD-162967/2021 |
| EPI_ISL_4301061 | hCoV-19/Italy/FVG-UD-162953/2021 |
| EPI_ISL_4301062 | hCoV-19/Italy/FVG-UD-162945/2021 |
| EPI_ISL_4301063 | hCoV-19/Italy/FVG-UD-162944/2021 |
| EPI_ISL_4327445 | hCoV-19/Italy/FVG-UD-199737/2021 |
| EPI_ISL_4301064 | hCoV-19/Italy/FVG-UD-199718/2021 |
| EPI_ISL_4301065 | hCoV-19/Italy/FVG-UD-188146/2021 |
| EPI_ISL_4301066 | hCoV-19/Italy/FVG-UD-188145/2021 |
| EPI_ISL_4301067 | hCoV-19/Italy/FVG-UD-196405/2021 |
| EPI_ISL_4301068 | hCoV-19/Italy/FVG-UD-194915/2021 |
| EPI_ISL_4301069 | hCoV-19/Italy/FVG-UD-177747/2021 |
| EPI_ISL_4301070 | hCoV-19/Italy/FVG-UD-209556/2021 |
| EPI_ISL_4301071 | hCoV-19/Italy/FVG-UD-200842/2021 |
| EPI_ISL_4301072 | hCoV-19/Italy/FVG-UD-209535/2021 |
| EPI_ISL_4327447 | hCoV-19/Italy/FVG-UD-209547/2021 |
| EPI_ISL_4301073 | hCoV-19/Italy/FVG-UD-208861/2021 |
| EPI_ISL_4301074 | hCoV-19/Italy/FVG-UD-200937/2021 |
| EPI_ISL_4301075 | hCoV-19/Italy/FVG-UD-208897/2021 |
| EPI_ISL_4301076 | hCoV-19/Italy/FVG-UD-209544/2021 |
| EPI_ISL_4301077 | hCoV-19/Italy/FVG-UD-200496/2021 |
| EPI_ISL_4301078 | hCoV-19/Italy/FVG-UD-199743/2021 |
| EPI_ISL_4301079 | hCoV-19/Italy/FVG-UD-199512/2021 |
| EPI_ISL_4301080 | hCoV-19/Italy/FVG-UD-192649/2021 |
| EPI_ISL_4301081 | hCoV-19/Italy/FVG-UD-208893/2021 |
| EPI_ISL_4301082 | hCoV-19/Italy/FVG-UD-200413/2021 |
| EPI_ISL_4301083 | hCoV-19/Italy/FVG-UD-208887/2021 |
| EPI_ISL_4301084 | hCoV-19/Italy/FVG-UD-208920/2021 |
| EPI_ISL_4301085 | hCoV-19/Italy/FVG-UD-208883/2021 |
| EPI_ISL_4301086 | hCoV-19/Italy/FVG-UD-208918/2021 |
| EPI_ISL_4301087 | hCoV-19/Italy/FVG-UD-209739/2021 |
| EPI_ISL_4301089 | hCoV-19/Italy/FVG-UD-198148/2021 |
| EPI_ISL_4301090 | hCoV-19/Italy/FVG-UD-200808/2021 |
| EPI_ISL_4301091 | hCoV-19/Italy/FVG-UD-200819/2021 |
| EPI_ISL_4301092 | hCoV-19/Italy/FVG-UD-200826/2021 |
| EPI_ISL_4301093 | hCoV-19/Italy/FVG-UD-200765/2021 |
| EPI_ISL_4301094 | hCoV-19/Italy/FVG-UD-200701/2021 |
| EPI_ISL_4301095 | hCoV-19/Italy/FVG-UD-200703/2021 |
| EPI_ISL_4301096 | hCoV-19/Italy/FVG-UD-200702/2021 |
| EPI_ISL_4301097 | hCoV-19/Italy/FVG-UD-202901/2021 |
| EPI_ISL_4301098 | hCoV-19/Italy/FVG-UD-203459/2021 |
| EPI_ISL_4301099 | hCoV-19/Italy/FVG-UD-210009/2021 |
| EPI_ISL_4301100 | hCoV-19/Italy/FVG-UD-202696/2021 |
| EPI_ISL_4301101 | hCoV-19/Italy/FVG-UD-201378/2021 |
| EPI_ISL_4327448 | hCoV-19/Italy/FVG-UD-202625/2021 |
| EPI_ISL_4327452 | hCoV-19/Italy/FVG-UD-202628/2021 |
| EPI_ISL_4301102 | hCoV-19/Italy/FVG-UD-200823/2021 |
| EPI_ISL_4301103 | hCoV-19/Italy/FVG-UD-200628/2021 |
| EPI_ISL_4301104 | hCoV-19/Italy/FVG-UD-200673/2021 |
| EPI_ISL_4301105 | hCoV-19/Italy/FVG-UD-204529/2021 |
| EPI_ISL_4301106 | hCoV-19/Italy/FVG-UD-202741/2021 |
| EPI_ISL_4301107 | hCoV-19/Italy/FVG-UD-202791/2021 |
| EPI_ISL_4301108 | hCoV-19/Italy/FVG-UD-203090/2021 |
| EPI_ISL_4301109 | hCoV-19/Italy/FVG-UD-202175/2021 |
| EPI_ISL_4301110 | hCoV-19/Italy/FVG-UD-202812/2021 |
| EPI_ISL_4301111 | hCoV-19/Italy/FVG-UD-200621/2021 |
| EPI_ISL_4301112 | hCoV-19/Italy/FVG-UD-198208/2021 |
| EPI_ISL_4301113 | hCoV-19/Italy/FVG-UD-201993/2021 |
| EPI_ISL_4301114 | hCoV-19/Italy/FVG-UD-197805/2021 |
| EPI_ISL_4301115 | hCoV-19/Italy/FVG-UD-208886/2021 |
| EPI_ISL_4301116 | hCoV-19/Italy/FVG-UD-209531/2021 |
| EPI_ISL_4301117 | hCoV-19/Italy/FVG-UD-195853/2021 |
| EPI_ISL_4301118 | hCoV-19/Italy/FVG-UD-195893/2021 |
| EPI_ISL_4301119 | hCoV-19/Italy/FVG-UD-195542/2021 |
| EPI_ISL_4301120 | hCoV-19/Italy/FVG-UD-177878/2021 |
| EPI_ISL_4301122 | hCoV-19/Italy/FVG-UD-192049/2021 |
| EPI_ISL_4301123 | hCoV-19/Italy/FVG-UD-181633/2021 |
| EPI_ISL_4301124 | hCoV-19/Italy/FVG-UD-195857/2021 |
| EPI_ISL_4327455 | hCoV-19/Italy/FVG-UD-201375/2021 |
| EPI_ISL_4301125 | hCoV-19/Italy/FVG-UD-195550/2021 |
| EPI_ISL_4301126 | hCoV-19/Italy/FVG-UD-183538/2021 |
| EPI_ISL_4301127 | hCoV-19/Italy/FVG-UD-192582/2021 |
| EPI_ISL_4301128 | hCoV-19/Italy/FVG-UD-200427/2021 |
| EPI_ISL_4301129 | hCoV-19/Italy/FVG-UD-200419/2021 |
| EPI_ISL_4301130 | hCoV-19/Italy/FVG-UD-200443/2021 |
| EPI_ISL_4301131 | hCoV-19/Italy/FVG-UD-208862/2021 |
| EPI_ISL_4327458 | hCoV-19/Italy/FVG-UD-188999/2021 |
| EPI_ISL_4301132 | hCoV-19/Italy/FVG-UD-186833/2021 |
| EPI_ISL_4301133 | hCoV-19/Italy/FVG-UD-194088/2021 |
| EPI_ISL_4301134 | hCoV-19/Italy/FVG-UD-195950/2021 |
| EPI_ISL_4301135 | hCoV-19/Italy/FVG-UD-208789/2021 |
| EPI_ISL_4301136 | hCoV-19/Italy/FVG-UD-195267/2021 |
| EPI_ISL_4301137 | hCoV-19/Italy/FVG-UD-195266/2021 |
| EPI_ISL_4301138 | hCoV-19/Italy/FVG-UD-195615/2021 |
| EPI_ISL_4301139 | hCoV-19/Italy/FVG-UD-197846/2021 |
| EPI_ISL_4301140 | hCoV-19/Italy/FVG-UD-201325/2021 |
| EPI_ISL_4301141 | hCoV-19/Italy/FVG-UD-195603/2021 |
| EPI_ISL_4301142 | hCoV-19/Italy/FVG-UD-196267/2021 |
| EPI_ISL_4301143 | hCoV-19/Italy/FVG-UD-195618/2021 |
| EPI_ISL_4301144 | hCoV-19/Italy/FVG-UD-194152/2021 |
| EPI_ISL_4301145 | hCoV-19/Italy/FVG-UD-194233/2021 |
| EPI_ISL_4301146 | hCoV-19/Italy/FVG-UD-192479/2021 |
| EPI_ISL_4301147 | hCoV-19/Italy/FVG-UD-196268/2021 |
| EPI_ISL_4301148 | hCoV-19/Italy/FVG-UD-196291/2021 |
| EPI_ISL_4301149 | hCoV-19/Italy/FVG-UD-194466/2021 |
| EPI_ISL_4301150 | hCoV-19/Italy/FVG-UD-216439/2021 |
| EPI_ISL_4301151 | hCoV-19/Italy/FVG-UD-193505/2021 |
| EPI_ISL_4301152 | hCoV-19/Italy/FVG-UD-202806/2021 |
| EPI_ISL_4301153 | hCoV-19/Italy/FVG-UD-202715/2021 |
| EPI_ISL_4301154 | hCoV-19/Italy/FVG-UD-202707/2021 |
| EPI_ISL_3047343 | hCoV-19/Italy/FVG-UD-201929/2021 |
| EPI_ISL_3055535 | hCoV-19/Italy/FVG-UD-201190/2021 |
| EPI_ISL_3047344 | hCoV-19/Italy/FVG-UD-202167/2021 |
| EPI_ISL_3047345 | hCoV-19/Italy/FVG-UD-201018/2021 |
| EPI_ISL_3047346 | hCoV-19/Italy/FVG-UD-201191/2021 |
| EPI_ISL_3047347 | hCoV-19/Italy/FVG-UD-201851/2021 |
| EPI_ISL_3047348 | hCoV-19/Italy/FVG-UD-199211/2021 |
| EPI_ISL_3047349 | hCoV-19/Italy/FVG-UD-202303/2021 |
| EPI_ISL_3047350 | hCoV-19/Italy/FVG-UD-204083/2021 |
| EPI_ISL_3047351 | hCoV-19/Italy/FVG-UD-202378/2021 |
| EPI_ISL_3047353 | hCoV-19/Italy/FVG-UD-202368/2021 |
| EPI_ISL_3047354 | hCoV-19/Italy/FVG-UD-201923/2021 |
| EPI_ISL_3047355 | hCoV-19/Italy/FVG-UD-198382/2021 |
| EPI_ISL_3047356 | hCoV-19/Italy/FVG-UD-202106/2021 |
| EPI_ISL_3047357 | hCoV-19/Italy/FVG-UD-202262/2021 |
| EPI_ISL_3047358 | hCoV-19/Italy/FVG-UD-200769/2021 |
