# Supplementary material for: Local occurrence and fast spread of B.1.1.7 lineage: A glimpse into Friuli Venezia Giulia
Source: PLoS One. 2021 Dec 14;16(12):e0261229. doi: 10.1371/journal.pone.0261229 (PMC8670677; doi:10.1371/journal.pone.0261229)
Supplement: Attachment — Submitted filename: PONE-D-21-25721-Recom.pdf [file pone.0261229.s003.pdf]

Summary of the research

The researcher group takes the opportunity to combine High Resolution Melting (HRM) approach and Next Generation Sequencing analysis (NGS) to screen the first four months of year and bring an overview to virus genomic evolution from January 1st 2021, until April 15th 2021 in this particular north-eastern region of Italy. The conclusion is an increasing of variant for this period as well as cluster sub-lineages with interest concern B.1.1.318 lineage.

-Keys list

Strengths

Sequenced samples number

Screening methods combination (HRM approach and sequencing)

Ethical guidelines respect

Spatio-temporal information of reported cases

Weaknesses:

Short duration of study (4 months)

Unuseful work in new mutation detection

No clinical signs data and demographic information of cases

No advices and perspectives to push interest of this paper

-Recommended course of action (major):

Introduction

-People speak about 19 A 20 A clades (line 40-41) without referring to Nextstrain nomenclature and its references (add website link and version).

-In addition, it needs to recall that Pangolin nomenclature system is not a way to "solve a problem" to GISAID or Nextstrain assignement supports. Pangolin is a complementary approach to other, and in especially to focus on the local dynamic and through lineages diversity instead of clades.

Conjugating nomenclature systems are so interesting method. Don't forgot also WHOs' (Alpha, Beta, etc..). It needs to be mentioned in the introduction too.

-Epidemiological context of pandemic in Italy and in this specific region prior of the study should be mentioned at this time point. This is to better understand the possible consequence in variants emergence and paper' interest.

Material and methods

-Precise the process of samples selection for 4 months: random n samples by months, no rule and only available samples?

-Add demographical and clinical information related to sequencing samples

-Give mor info in High resolution melting (HRM) technic and its benefices: melting temperature analysis...

-Notice date of assignement by using Nextstrain and Pangolin (line 101 103) due to update in continuous

Results

-Lines 108- 115 need to be moved to in material ad methods

-Data shows only lineages reports (Pangolin assignement). Its of Nextstrain assignement miss and added concerning 1508 seq (i.e.: is it clade 20Ia and for all?)

-Figure 2: most of lineage are B.1.1.7 lineages (major %). but what about the % remaining?

- Figure 1: It shows delay between infected patients (blue line) and hospitalizations number (green line). In addition, it should be possible to link the increasing in variants incidence to specific clinical signs changes and/or demographical population targeted (ages, sex etc..) for 4 months at hospital (i.e: table with progression index, age, sex, etc...). It provides opportunity to perform additionnal multivariable statistical analysis in correlation between to these events.
-In contrast, work in new mutations detection has not to be place on main document. It makes confusing analysis without arguing in interested to support main message. This part may be rewrited by only speaking in known mutations concern to variant leading to review figure 1 C/D and Table 2 to suppl.

Discussion

Arguing in benefices to combine HRM + sequencing approaches, or oppose to it, as a time-consuming as well as too restrictive (no new variants discovery) approach compared to directly perform sequencing of all positive samples. NGS allows to open the opportunity to detect other variants here, already described in worldwide and to frontiers as well as to propose under monitored variants as concern. Moreover, it is surprising to only detect 2 lineages B.1.1.7 and B.1.1.318. In general, SARS-Cov-2 sequencing in other countries shows diversity in lineages more and even in clades. This poorness is it justify or not (among >1500 seq.)? No detection of parental lineages (i.e: B.1) ?
-Speak in possible causes of variant introduction at the beginning of year by providing information to reported cases and/or to frontiers regions (business exchanges, traveler, unlock-down event, vacancy…).
-In line 50, people mention "Viral genome sequencing coupled to epidemiological surveillance is mandatory", it is a pity that the sequencing is not associated to epidemiological during the study period to discuss on it here.

Conclusion

No advice and/or perspectives to use sequencing at hospital (to management) as well at frontiers to target new variant introductions in this strategic part of the Italian region.

Graphics

-Table 1: this relationship between GISAIS/Nextstrain and Pangolin nomenclature systems is well described in Alma et al, (https://doi.org/10.2807/1560-7917.ES.2020.25.32.2001410) . Paper could be noticed to remove table 1.
Or table1 should be redesigned (Column 1 GISAID, Column 2 Nextstrain, Colum 3 Pangolin) and put on suppl. In addition, table 1 needs more legend details (choice of some variants and not others, mutations code (p.?) and also mentioned WHO nomenclature (alpha, beta etc..).
-Figure 1
1A: 4 maps, one for each month, allow to localize positive cases in space (in green), identify some of them associated to variant (in black) and possibly show cluster emergence in time (i.e. radiation or centralization).
1B: it is related to table 3 and better to put together one above other.
1C and 1D are structural and mutations concern: to put apart in a new figure, especially to suppl. as already recommanded.

-Recommended course of action (minor):

Introduction

it may be important to justify the neutral genetic drift (line 33) and enhance of viral fitness affect in mutations constellations (line 53) with SARS-Cov-2 references.

Methods

% of targeted coverage, number of sequencing with >10% missing genome (no success).

Results

Bring information in patient treatments and management results to variants emergence at hospital. It might affect the quality of diagnostic and/or sequencing success?!

Discussion

The emergence of variant is it associate to guidelines changes in local regions such as lock-down, diagnostic or vaccine recommendation?

Remove 188-195 lines as losing the lector to main goal

Figure

Improve the resolution quality of figures

Overview of the manuscript's

On real-time screening is the cornerstone strategy to fight against current Covid pandemic, especially variants sequencing. Variants increase transmissibility, virulence. Their association to fatal diseases progression justifies the relationship between variants progression and the hospitalization cases number in this paper. Is the detection of new mutations important, here? Giving more details in the clinical signs of patients (with B.1.1.7 detection), in lineages diversity in this region and at frontiers should be benefices to paper.
